# LPA_1_ antagonist-derived LNPs deliver A20 mRNA and promote anti-fibrotic activities

**DOI:** 10.1007/s12274-024-6747-6

**Published:** 2024-06-27

**Authors:** Jingyue Yan, Diana D. Kang, Chang Wang, Xucheng Hou, Shi Du, Siyu Wang, Yonger Xue, Zhengwei Liu, Haoyuan Li, Yichen Zhong, Binbin Deng, David W. McComb, Yizhou Dong

**Affiliations:** 1 Division of Pharmaceutics & Pharmacology, College of Pharmacy, The Ohio State University, Columbus, OH 43210, USA; 2 Icahn Genomics Institute, Precision Immunology Institute, Department of Immunology and Immunotherapy, Department of Oncological Sciences, Tisch Cancer Institute, Biomedical Engineering and Imaging Institute, Friedman Brain Institute, Icahn School of Medicine at Mount Sinai, New York, NY 10029, USA; 3 Center for Electron Microscopy and Analysis, The Ohio State University, Columbus, OH 43212, USA; 4 Department of Materials Science and Engineering, The Ohio State University, Columbus, OH 43210, USA

**Keywords:** lysophosphatidic acid receptor 1 (LPA_1_) antagonist, tumor necrosis factor *α*-induced protein 3 (A20), lung fibrosis, lipid nanoparticles, mRNA

## Abstract

Activated fibroblasts are major mediators of pulmonary fibrosis. Fibroblasts are generally found in the connective tissue but upon activation can generate excess extracellular matrix (ECM) in the lung interstitial section. Therefore, fibroblasts are one of the most targeted cells for treating idiopathic pulmonary fibrosis (IPF). Here, we develop an anti-fibrotic platform that can modulate both the lysophosphatidic acid receptor 1 (LPA_1_) and the inflammatory pathway through tumor necrosis factor *α*-induced protein 3 (TNFAIP3, also known as A20) in fibroblasts. First, we synthesized a series of LPA_1_ antagonists, AM095 and AM966, derived amino lipids (LA lipids) which were formulated into LA-lipid nanoparticles (LA-LNPs) encapsulating mRNA. Specifically, LA5-LNPs, with AM966 head group and biodegradable acetal lipid tails, showed efficient A20 mRNA delivery to lung fibroblasts *in vitro* (80.2% ± 1.5%) and *ex vivo* (17.2% ± 0.4%). When treated to primary mouse lung fibroblasts (MLF), this formulation inhibited fibroblast migration and collagen production, thereby slowing the progression of IPF. Overall, LA5-LNPs encapsulated with A20 mRNA is a novel platform offering a potential approach to regulate fibroblast activation for the treatment of IPF.

## 1 Introduction

Idiopathic pulmonary fibrosis (IPF) is a chronic condition marked by an abnormal buildup of extracellular matrix (ECM) in the lung interstitial section, leading to pulmonary dysfunction [1, 2]. Globally, approximately 3 million people are affected by IPF with a median survival duration of less than 5 years if left untreated [3]. Although the precise cause of IPF is undetermined, it has been linked to different factors including environmental exposure, family history, infections, as well as medication [4]. The mechanism of IPF development is complicated, but immune cells and inflammatory signals are believed to play a role in the pathogenesis and progression of fibrosis [5]. Due to challenges in precise diagnosis, poor prognosis, limited medication options, and high mortality, developing new treatment regimens for IPF has reached unprecedented interest levels. Currently, only two anti-fibrotic medications are approved for IPF treatment by the US Food and Drug Administration (FDA), Pirfenidone and Nintedanib. However, both drugs showed notable side effects and are only palliative [6]. Therefore, the development of novel treatments to slow down or possibly reverse the IPF progression is of urgent need.

The pathogenesis of IPF starts from lung epithelial microinjuries caused by the aforementioned factors, triggering abnormal healing processes and immune activation, which leads to excessive myofibroblast activation and proliferation [7]. Therapeutic agents have been developed to suppress immune activation and fibroblast proliferation by targeting one of these steps in the pathogenesis of IPF [1]. Both Pirfenidone and Nintedanib function by targeting fibroblasts, which are the primary effector cells that engender the development of IPF. While pirfenidone exerts anti-inflammatory effects and acts as an antioxidant, Nintedanib inhibits the platelet-derived growth factor (PDGF) receptor which reduces the chemotaxis and proliferation of myofibroblasts [8, 9]. Another proposed mechanism of modulating fibroblasts is through the lysophosphatidic acid receptor 1 (LPA_1_), which is a G protein-coupled receptor (GPCR) that binds to lysophosphatidic acid (LPA) [10]. Evidence suggests that LPA_1_ plays an essential role in modulating wound healing by stimulating chemotaxis through fibroblast recruitment [11]. AM095 and AM966, two potent LPA_1_ antagonists, have demonstrated efficacy in reducing tissue injury, inflammation, and fibrosis in IPF mouse models [12, 13].

Aberrant immune responses have been associated with the pathophysiology of autoimmune and inflammatory disorders. The pleiotropic ubiquitin-modifying enzyme A20, encoded by the tumor necrosis factor alpha-induced protein 3 (*Tnfaip3*) gene, has attracted much attention for its anti-inflammatory signaling that closely regulates multiple immune cell functions in diverse human fibrotic diseases [14]. Studies have reported that A20 regulates several inflammatory signaling cascades, notably the canonical nuclear factor-*κ*B (NF-*κ*B) signaling pathway [14]. The suppression of A20 enzymatic activities has been shown to play a role in the advancement of lung fibrosis, making A20 a potential target in IPF treatments [15, 16].

**Figure 1:**
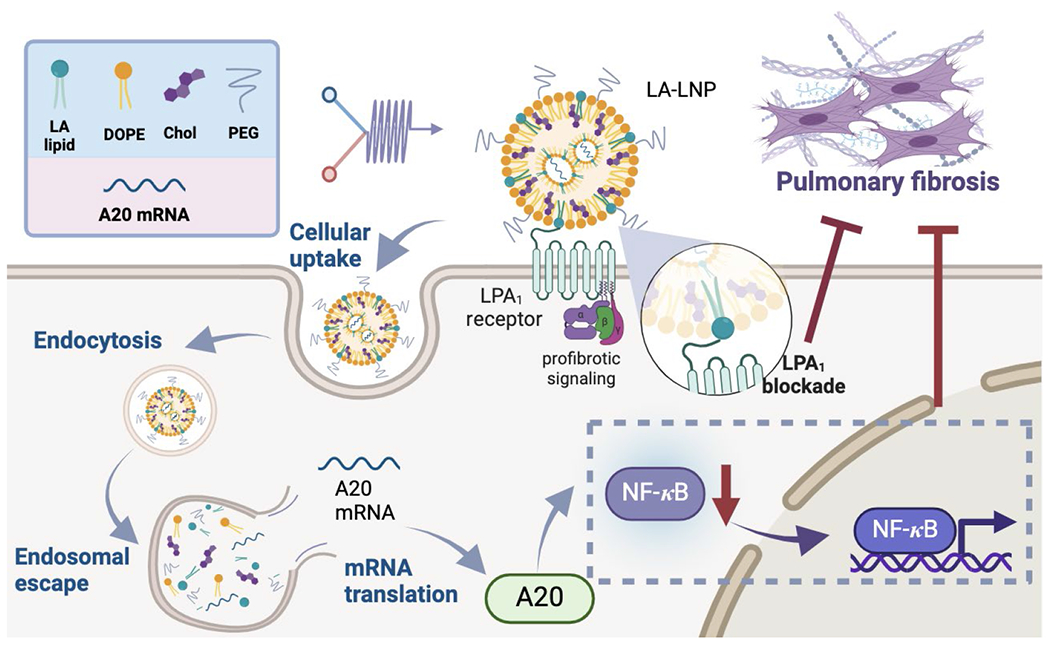
Schematic illustration of LA5 A20-LNPs mitigate fibrosis progression. The LA5-LNP is recognized by the upregulated LPA_1_ receptors on activated fibroblasts, facilitating efficient A20 mRNA delivery to fibroblasts as well as blocking the LPA_1_ receptor-mediated profibrotic pathways. The A20 mRNA is then translated into A20 protein in the cytoplasm, which negatively regulates NF-*κ*B activation, leading to a slower progression of pulmonary fibrosis.

Based on previous findings, we hypothesize that the integration of LPA_1_ antagonist mediated anti-fibrotic activities with A20 mediated anti-inflammatory activities may suppress fibroblast activation, and thereby limit the fibrotic responses and IPF development. Lipid nanoparticles (LNPs) exhibit significant potential in exogenous gene expression through the delivery of mRNA, thus presenting a promising approach to transiently elevate A20 levels in lung fibroblasts [17–25]. To explore this strategy, we synthesized LPA_1_ antagonists (AM095 and AM966)-derived amino lipids (LA lipids) and formulated them into LNPs (LA-LNPs) for A20 mRNA delivery to activated fibroblasts (Fig. 1). Our results showed that LA5-LNPs, with AM966 as the head group and branched biodegradable acetal lipid tails, can efficiently deliver A20 mRNA to lung fibroblasts both *in vitro* and *ex vivo*. This approach effectively inhibits fibroblast proliferation, migration, and collagen synthesis in primary mice lung fibroblasts (MLF). This study underscores the potential of integrating multiple anti-fibrotic pathways for IPF treatment.

## 2 Results and discussion

**Figure 2:**
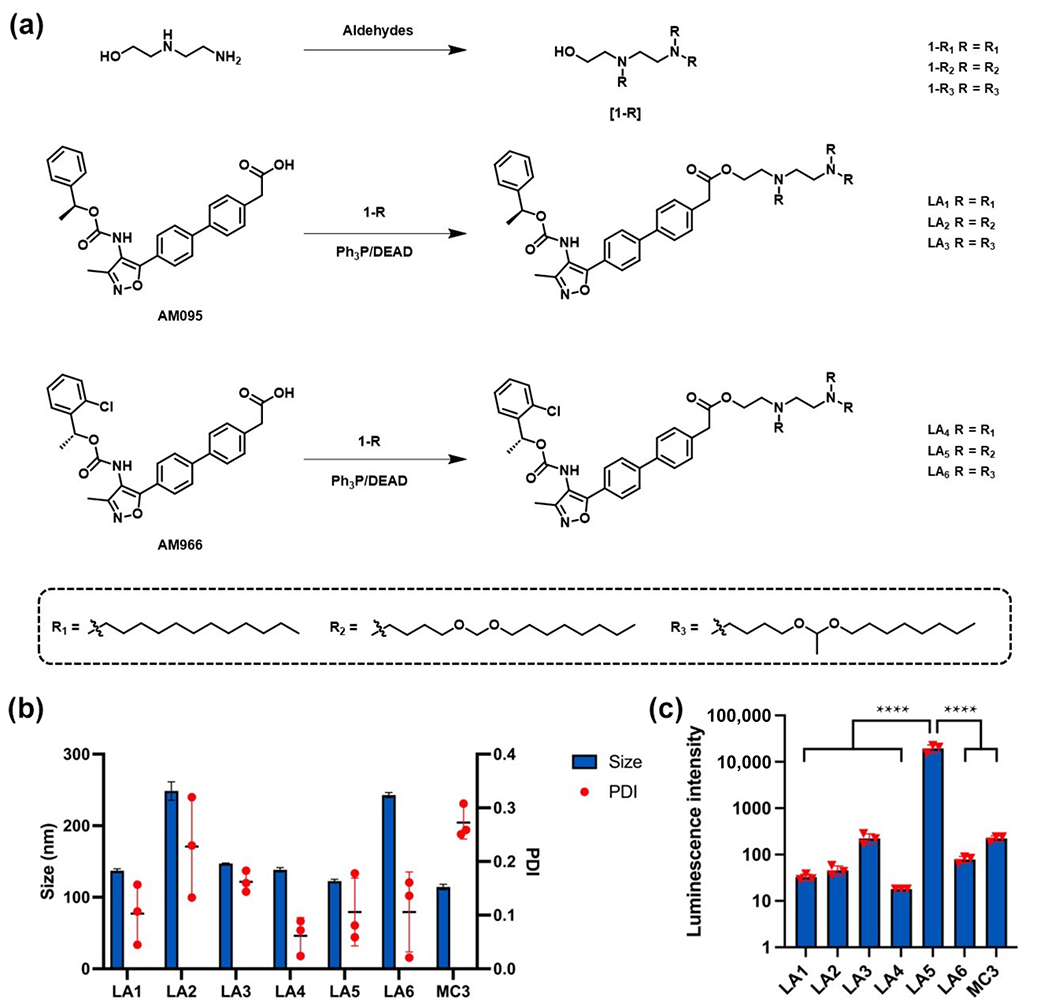
Synthesis of LPA_1_-antagonist derived lipids (LAs) and characterization of LA-LNPs. (a) Synthetic routes to LA1–LA6. (b) Size and PDI of LA-LNPs and MC3-LNPs encapsulated with FLuc mRNA. LA-LNPs were formulated at the same lipid molar ratio of LA:DOPE:Chol:PEG = 20:30:40:0.75. (c) Luminescence intensity in MLg cells. Data in (b) and (c) are presented as the mean ± S.D. (*n* = 3). Statistical significance in (c) is analyzed by one-way ANOVA with Dunnett’s multiple comparison test. ****P < 0.0001.

To produce LPA_1_ antagonist (AM095 and AM966)-derived amino lipids (LA lipids), we first synthesized three different hydroxylated ionizable lipids tails with either saturated carbon chains or bioresponsive acetal-containing chains [19]. These hydroxylated ionizable lipids were then coupled with AM095 and AM966 using Mitsunobu esterification to generate LA lipids. LA lipids consist of LPA_1_ antagonist-based heads, amino cores, and various lipid tails (Fig. 2(a)). The structures of LA lipids were validated using ^1^H nuclear magnetic resonance (NMR) and mass spectrometry (shown in the Electronic Supplementary Material (ESM)).

The synthesized LA lipids were then formulated with the addition of 1,2-dioleoyl-sn-glycero-3-phosphoethanolamine (DOPE), cholesterol (Chol), and DMG-PEG_2000_ (PEG) to obtain LA-LNPs. To test the mRNA delivery efficiency, the different LA - LNPs were formulated with firefly luciferase (FLuc) mRNA and then treated to MLg cells, a lung fibroblast cell line [19, 26–28]. All formulated LA-LNPs have a hydrodynamic diameter under 300 nm with a polydispersity index (PDI) below 0.3 (Fig. 2(b)). FLuc mRNA encapsulated in LA5-LNPs showed over a 100-fold higher luminescence intensity compared to that of the other LA-LNPs, including the D-Lin-MC3-DMA (MC3) lipid formulation utilized in the FDA-approved siRNA-LNP therapy, ONPATTRO^®^ (Fig. 2(c)) [29]. The luminescence intensity serves as an indicator for mRNA delivery efficiency, which can be analyzed to evaluate the structure-activity relationship of LA lipids for mRNA delivery. Lipids derived from two distinct LPA_1_ antagonists, AM095 and AM966, exhibited varying luminescence intensities in MLg cells, suggesting that the headgroup properties of LA lipids may affect mRNA delivery efficiency. Meanwhile, the lipid tail structure can also alter the mRNA delivery efficiency. For example, LA1- and LA4-LNPs with fully saturated 12 hydrocarbon chain yields lower mRNA delivery efficiency compared to lipid tails with an acetal group. The enhanced delivery efficiency could stem from variations in the critical packing parameters of the lipid tails [28]. The acetal groups may facilitate the formation of the hexagonal phase upon acidification in the endosome to promote endosomal escape of the mRNA. Notably, LA5 with an AM096 head group and three tails containing formaldehyde acetal groups exhibited over 100-fold greater luminescence intensity than MC3. These findings indicate that the mRNA delivery efficiency of LA lipids is significantly influenced by both the headgroup characteristics and the functional groups in the lipid tails.

**Figure 3:**
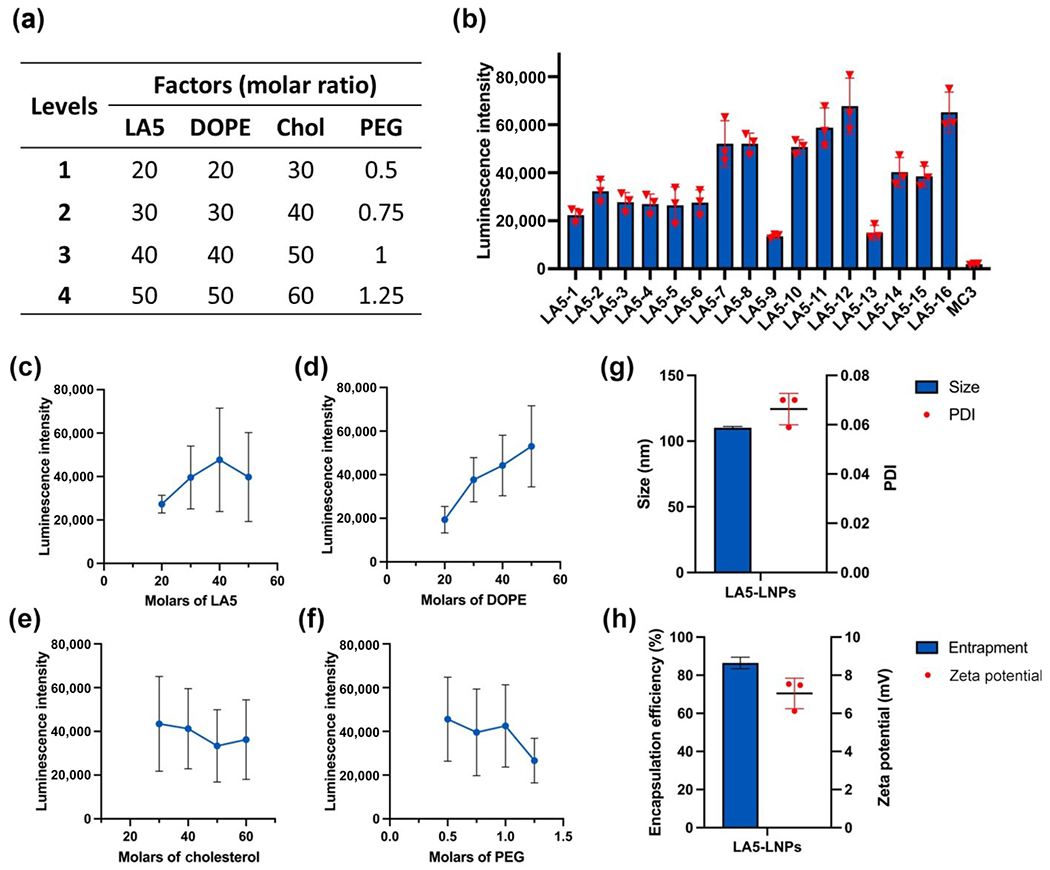
Orthogonal optimization and characterization of LA5-LNPs. (a) Orthogonal optimization table with 4 levels for each lipid. (b) Luminescence intensity after the delivery of FLuc mRNA in 16 formulated LA5-LNPs with different lipid compositions. Impact trend of lipid component (c) LA5, (d) DOPE, (e) cholesterol, and (f) PEG in LA5-LNPs. The optimized LA5-LNP has a molar ratio of LA5:DOPE:Chol:PEG = 40:50:30:0.5. (g) Size and PDI of optimized LA5-LNPs carrying FLuc mRNAs. (h) Encapsulation efficiency and zeta potential of LA5-LNPs carrying FLuc mRNAs. Data in (b)–(h) are presented as the mean ± S.D. (*n* = 3).

To further optimize the formulation of LA5, we conducted a design of experiment (DoE) based on the L_16_ (4)^4^ orthogonal table and obtained 16 LA5 FLuc-LNPs formulations with different lipid molar ratios (Fig. 3(a), and Table S2 in the ESM) [26]. Luminescence intensity was measured after the delivery of FLuc mRNA in 16 formulated LA5-LNPs in MLg cells (Fig. 3(b)). Four levels of each component were plotted based on the observed luminescence intensity shown in Figs. 3(c)–3(f). The trend of LA5 showed peak luminescence intensity at a molar ratio of 40, so we increased the molar ratio of LA5 from 20 to 40 (Fig. 3(c)). As shown in Fig. 3(d), increased DOPE levels facilitated mRNA delivery efficiency, therefore we increased the molar ratio of DOPE from 30 to 50. The levels of Chol and PEG were decreased from 40 to 30, and 0.75 to 0.5 based on the trends shown in Figs. 3(e) and 3(f), respectively. The top formulation of lipid molar ratios based on the DoE optimization is LA5:DOPE:Chol:PEG = 40:50:30:0.5. The optimized LA5 LNPs result in a comparable delivery efficiency to LNPs formulated with ALC-0315 lipid, which is used for the Pfizer/BioNTech COVID-19 vaccine (Fig. S1(a) in the ESM).

**Figure 4:**
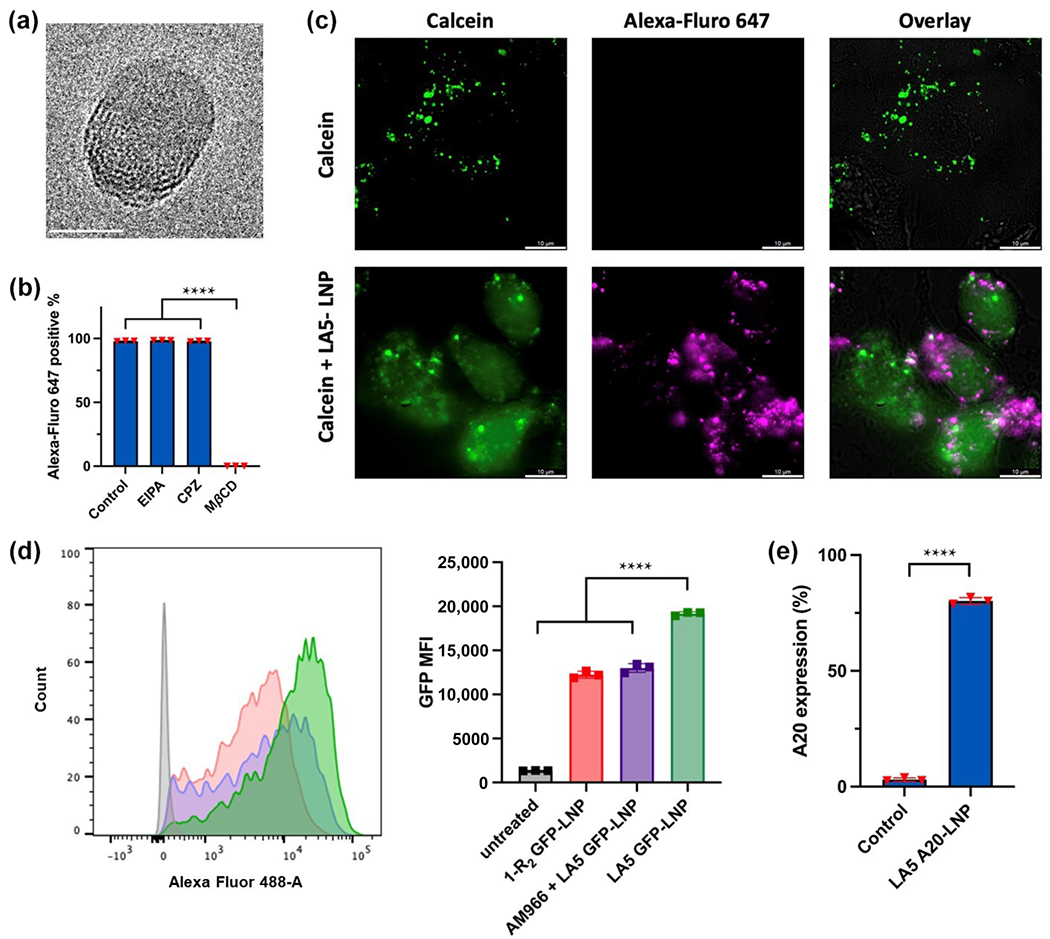
LA5-LNPs mediated mRNA delivery to lung fibroblasts. (a) Cryo-TEM characterization of LA5-LNPs (scale bar = 50 nm). (b) MLg cell uptake of LA5-LNPs containing Alexa-Fluor 647 RNAs was investigated with endocytosis inhibitors, EIPA, CPZ, and M*β*CD. (c) Confocal microscopy images of MLg cells incubated with calcein, with or without LA5-LNPs (scale bars = 10 μm). (d) Mean GFP intensity after the treatment with LA5-LNPs carrying GFP mRNA. (e) LA5-LNPs mediated delivery of A20 mRNA in MLg cells. Data in (b), (d), and (e) are presented as the mean ± S.D. (*n* = 3). Statistical significance in (b) and (d) are analyzed by one-way ANOVA with Dunnett’s multiple comparison test. Statistical significance in (e) is analyzed by student’s t test. ****P < 0.0001.

We then characterized the physiochemical properties of the optimized formulation. The LA5-LNPs have a particle size of 110.1 ± 1.1 nm with a PDI of 0.066 ± 0.006 (Fig. 3(g)). The particles have an RNA encapsulation rate of around 86.4% ± 3.0%, and they were positively charged at around 7.0 mV in a lightly acidic formulation buffer (Fig. 3(h)). Images from cryogenic transmission electron microscopy (cryo-TEM) showed that the LA5-LNPs have an elliptical structure (Fig. 4(a)). Once endocytosed into the cell, the LA5-LNPs encapsulated mRNA needs to escape from the endosomes to reach the cytoplasm for translation into corresponding proteins. To study the internalization mechanisms of LA5-LNPs, Alexa-Fluor 647 (AF647) labeled RNA were encapsulated in the LNPs. Before the LNPs were administered, pre-treatment with 5-(N-Ethyl-N-isopropyl) amiloride (EIPA), chlorpromazine (CPZ), and methyl-*β*-cyclodextrin (M*β*CD) were conducted to inhibit the macropinocytosis, clathrin mediated, and caveolae endocytic pathways, respectively. Cells pre-treated with M*β*CD significantly blocked LA5-LNP uptake, indicating that MLg cells primarily internalized LA5-LNPs through the caveolae-mediated endocytic pathway (Fig. 4(b)). To evaluate if the RNA can escape from the endosome, cells were co-incubated with LA5 AF647-LNPs together with calcein, a membrane-impermeable fluorophore that is typically retained in the endosome. We observed that while the cells treated with calcein alone showed punctate green-fluorescent signals in the endosome, the cells treated with both the calcein and LA5 AF647-LNPs showed diffused green-fluorescent signals throughout the cytoplasm, suggesting endosomal membrane rupture and subsequent release of AF647 RNA into the cytoplasm (Fig. 4(c)).

To assess whether the LA5-LNPs can target lung fibroblasts with enhanced LPA_1_ receptor expression, we treated MLg cells with 1-R_2_ GFP-LNPs, where 1-R_2_ is LA5 without AM966 conjugation, or added excess amount of AM966 (10 μM) to saturate and block the LPA_1_ receptor on lung fibroblast before adding LA5 GFP-LNPs. We observed that groups with no LPA_1_ targeting (1-R_2_ GFP-LNPs) or LPA_1_ blockage showed lower green fluorescent protein (GFP) intensity compared to LA5 GFP-LNPs, indicating that AM966 conjugation in LA5-LNPs could increase particle uptake in lung fibroblasts, mediated through the LPA_1_ interaction (Fig. 4(d)). To assess whether we can increase A20 expression levels in lung fibroblasts, we delivered A20 mRNA using LA5-LNPs and found significantly higher A20 expression in the MLg cell line (80.2% ± 1.5%) compared to PBS treated cells (3.0% ± 0.8%) (Fig. 4(e)).

**Figure 5:**
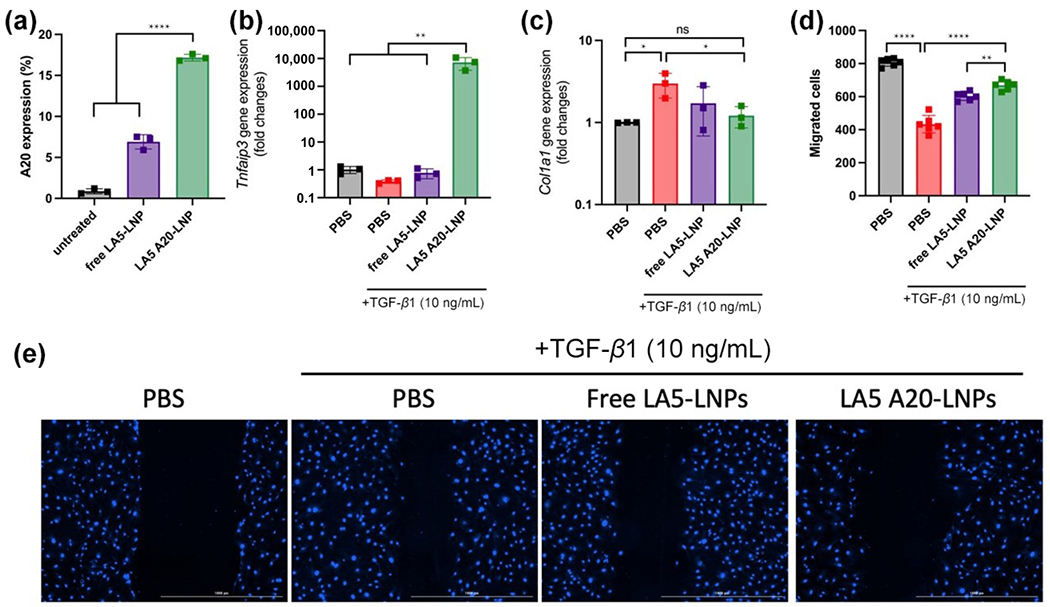
Delivery of A20 mRNA by LA5-LNPs ameliorates fibroblast activation by reducing collagen synthesis and abating fibroblast migration in primary mice lung fibroblasts. (a) LA5-LNPs mediated A20 mRNA delivery in MLF cells. (b) *Tnfaip3* and (c) *Col1a1* gene expression levels after the treatment with TGF-*β*1 as determined by qRT-PCR. (d) Statistical data and (e) representative images of wound-healing assay in MLFs treated with PBS, free LA5-LNPs, or LA5 A20-LNPs (*n* = 6). Scale bars: 1 mm. Data in (a)–(d) are presented as the mean ± S.D. (*n* = 3). Statistical significance in (a)–(d) is analyzed by one-way ANOVA with Dunnett’s multiple comparison test. *P < 0.05; ns: not significant.

Next, we investigated the safety, delivery efficiency, and anti-fibrotic functions of LA5 A20-LNPs in isolated primary mouse lung fibroblasts (MLF). Compared to other FDA-approved LNPs formulations based on ALC-0315 or MC3 ionizable lipids, LA5 LNPs showed comparable cytotoxic profiles as measured by an MTT assay (Fig. S1(b) in the ESM). As depicted in Fig. 5(a), LA5 A20-LNPs notably elevated A20 protein expression in MLF (17.2% ± 0.4%), 20-fold of that of PBS (0.9% ± 0.3%) and 2-fold of free LA5-LNPs without A20 mRNA encapsulation (6.9% ± 0.9%). We found free LA5-LNPs without A20 mRNA encapsulated could also increase A20 protein expression to some extent. We speculate this is due to the suppression of LPA_1_ mediated profibrotic pathway, which can restore the activities of A20 in lung fibroblasts. Fibroblasts can differentiate into a myofibroblast phenotype, often characterized by the overexpression of *α*-smooth muscle actin (*α*-SMA) upon stimulation with TGF-*β*1 [30]. Upon myofibroblast differentiation, fibroblasts become proliferative, migratory, and increase the production of ECM components, such as collagen. Therefore, we further investigated if LA5 A20-LNPs could inhibit TGF-*β*1-induced A20 downregulation and myofibroblast differentiation in MLFs [15]. MLFs were initially pre-treated with TGF-*β*1, followed by treatment with PBS, free LA5-LNPs, or LA5 A20-LNPs. While TGF-*β*1 treatment caused the suppression of *Tnfaip3* gene expression level, LA5 A20-LNPs could significantly increase *Tnfaip3* gene expression in MLFs to exert its anti-fibrotic activities (Fig. 5(b)). The anti-fibrotic activity is demonstrated by the reduced *Col1a1* mRNA levels, where TGF-*β*1 treatment alone increased collagen synthesis while LA5 A20-LNPs treatment reduced it to inactivated levels (Fig. 5(c)). Furthermore, we evaluated the effect of LA5 A20-LNPs on fibroblast migration using an *in vitro* scratch assay, which was conducted by creating a straight-line scratch across the MLF monolayer. The fibroblast migration was significantly decelerated in the LA5 A20-LNP group compared to the other two groups treated with TGF-*β*1 (Figs. 5(d) and 5(e)).

## 3 Conclusions

Here, we have developed a platform to slow down IPF progression using LA5-LNPs. As the LPA_1_ receptor is highly expressed on fibroblast cell surface membrane, the lipidized LPA_1_ antagonist in the LA5-LNP formulation enhances mRNA uptake in lung fibroblasts and reduces chemotaxis and proliferation of lung fibroblast upon LPA_1_ blockade. On the other hand, A20 plays a significant role in the downregulation of fibrotic responses by suppressing the NF-*κ*B signaling pathway [16]. We observed a strong effect on blocking the LPA_1_ signaling pathway and restoring A20 enzymatic activities. Our findings indicate that LA5-LNPs encapsulated with A20 mRNA exhibit stronger antifibrotic activities compared to free LA5-LNPs in mice lung fibroblasts, as evidenced by a slower migration process and reduced collagen synthesis. The precise targeting of lung tissues *in vivo* is important for the effective utilization of LA5 A20-LNPs in the treatment of pulmonary fibrosis. Local administration methods, such as intratracheal injection, oropharyngeal aspiration, and intranasal delivery, can direct LNPs specifically to the lung [31–33]. Furthermore, the manipulation of the LNPs formulation, whether through the design of the ionizable lipid or modulating the charge characteristics of LNPs, can tune the LNP tropism towards specific organs after systemic administration [34, 35]. For instance, the incorporation of a cationic lipid, such as dioleoyl-3-trimethylammonium propane (DOTAP), within the LNP formulation has been demonstrated to enable selective mRNA delivery to the lungs [34]. These approaches may be applied to LA5-LNPs for future *in vivo* studies to advance the therapeutic potential of LA5-LNPs in pulmonary fibrosis. In summary, our findings provide proof of concept that effective delivery of A20 mRNA with LA5-LNPs can mitigate fibrotic activities in IPF. We believe that with further examinations of *in vivo* delivery strategies and of safety profiles, the combination of LPA_1_ antagonist and A20 pathway holds great promise as a potential treatment for IPF.

## 4 Experimental

### 4.1 Materials

All chemicals and solvents were purchased from Fisher Scientific unless otherwise listed. AM095 and AM966 were obtained from MedChemExpress (NJ, USA). DOPE was purchased from Avanti Polar Lipids (AL, USA). DMG-PEG2000 was purchased from NOF America Corporation (NY, USA).

### 4.2 Cell lines and maintenance

MLg [Mlg 2908] (ATCC^®^ CCL-206^™^) cells were purchased from American Type Culture Collection (ATCC, VA, USA), and cultured in Eagle’s Minimum Essential Medium (Thermo Fisher, MA, USA) containing 10% fetal bovine serum (Invitrogen, MA, USA).

### 4.3 Isolation of primary mouse lung fibroblast (MLF)

MLF was isolated from C57BL/6J mice and cultured in DMEM containing 10% FBS and 1% penicillin-streptomycin. In brief, the lung was finely chopped then enzymatically digested in serum-free Dulbecco’s modified Eagle’s medium (DMEM) supplemented with Liberase (Roche, Switzerland) and 1% penicillin-streptomycin for 30 min at 37 °C. Digestion was terminated by adding a DMEM medium with 10% fetal bovine serum (FBS). MLFs were cultured for 3 days before being passaged. MLFs were treated with TGF-*β*1 (R&D Systems, USA) at a concentration of 10 ng/mL in DMEM medium to induce myofibroblast differentiation.

### 4.4 Characterization of nanoparticle formulation

mRNA encapsulated LNPs were formulated with LAs and helper lipids DOPE, cholesterol, 1,2-dimyristoyl-rac-glycero-3-methoxypolyethylene glycol-2000 (DMG-PEG_2000_). All lipid components were dissolved in ethanol at desired concentrations, while mRNA was dissolved in aqueous citrate buffer. The ethanol and aqueous phases were mixed rapidly together at a volume ratio of 1:3 (10:1 weight ratio of LA:mRNA) using a rapid nanomedicine system INano L + microfluidics instrument from Micro & Nano Biologics Technology Ltd. The N/P ratio of the formulated LA-LNPs is shown in Table S1 in the ESM. The mRNA encapsulated LNPs underwent an 80-min dialysis in PBS buffer using Slide-A-Lyzer Dialysis Cassettes (Life Technologies, NY, USA) and were then filtered through 0.22 μm polyethersulfone (PES) filter (Millipore Sigma, MA, USA). The particle size and zeta potential of LA-LNPs were determined using Zetasizer NanoZS (Malvern, UK). Additionally, the mRNA encapsulation efficacy (*EE*%) was determined by Quant-it^™^ RiboGreen RNA Assay (ThermoFisher Scientific, MA, USA) [26]. The morphology of LA5-LNPs was assessed using a Glacios Cryo-TEM device (Thermo Fisher Scientific, MA, USA) using the methods described previously [28]. For the luminescence readout, a dose of 50 ng FLuc RNA encapsulated in LA-LNPs was treated to MLg cells, and luminescence readout was conducted after 18 h of co-culture.

### 4.5 Endosomal escape

A total of 6 × 10^4^ MLg cells in 300 μL of complete medium were plated in each chamber of the imaging dish (Ibidi USA Inc, WI, USA) and cultured overnight at 37 °C with 5% CO_2_. Calcein with or without LA5-LNPs containing Alexa-Fluro 647 RNA were added to each chamber. Cells were washed twice with PBS after 2 h of co-incubation, and imaged using Leica DMi8 Brightfield (Leica, Germany).

### 4.6 A20 mRNA preparation

The A20/Tnfaip3 gene cDNA sequence was retrieved from GenBank (Reference number: U19463.1). Linearized A20 dsDNA sequence was acquired from IDT and integrated into pUC19 vector containing T7 promoter and optimized UTRs using NEBuilder^®^ HiFi DNA Assembly (New England Biolabs, MA, USA), and the correct plasmid was confirmed by sanger sequencing. The mRNA was enzymatically synthesized following a previously published protocol [22].

### 4.7 *In vitro* and *ex vivo* delivery of A20 mRNA to lung fibroblasts

We evaluated the delivery of A20 mRNA to MLg cells or MLF using FITC-labeled anti-A20/TNFAIP3 monoclonal antibody (Novus Biologicals^™^, Cat# NBP177533F) via flow cytometry. Initially, 1 × 10^5^ MLg or MLF cells were seeded in a 24-well plate and incubated at 37 °C in 5% CO_2_ incubator for 24 h. Subsequently, the cells were treated with LA5-LNPs containing 500 ng A20 mRNAs for 18 h. Following the treatment, the cells underwent fixation and permeabilization using eBioscience^™^ Intracellular Fixation & Permeabilization Buffer Set (ThermoFisher, MA, USA). The cells were then incubated with fluorescein isothiocyanate (FITC) labeled anti-A20/TNFAIP3 monoclonal antibody (clone 59A426, 1:50 dilution) in cold PBS containing 1% FBS for 30 min at 4 °C. Cellular uptake was subsequently assessed using a BD LSR Fortessa flow cytometer.

### 4.8 *In vitro* scratch assay

1.2 × 10^5^ MLFs were seeded per well in a clear 12-well plate and cultured in DMEM containing 10% FBS and 1% penicillin-streptomycin overnight at 37 °C. MLFs were treated with LA5-LNP with or without A20 mRNA in the presence of 10 ng/mL TGF-*β*1, followed by incubation at 37 °C for 24 h. A line was drawn on the cell monolayer using a sterile 200 μL pipette tip and images of each well were captured after 6 h incubation in serum-free medium using the BioTek Cytation 5 Cell Imaging Multimode Reader (Agilent, USA).

### 4.9 Quantitative reverse transcription-polymerase chain reaction (RT-PCR)

Total RNA was extracted from MLF with RNeasy kit (Qiagen, USA). Subsequently, cDNA was synthesized using Superscript^™^ IV VILO^™^ Master Mix with ezDNase (Invitrogen, USA). For qRT-PCR gene expression analysis, TaqMan^™^ Fast Advanced Master Mix for qPCR (Applied Biosystems^™^, USA) was utilized with TaqMan^™^ Gene Expression Assay ID Mm00437121_m1 for mouse *Tnfaip3*, Mm00801666_g1 for *Col1a1* (Applied Biosystems^™^, USA) on a QuantStudio^™^ 6 Pro Real-Time PCR system (Applied Biosystems^™^, USA). qRT-PCR data were normalized to *Gapdh* analyzed with TaqMan^™^ Gene Expression Assay ID Mm99999915_G1 as a housekeeping gene standard. Fold changes of target mRNAs were analyzed using the 2^−ΔΔCT^ method.

### 4.10 Data analysis

No collected experimental data were excluded for the quantitative analysis. The number of repetitions in each group and the error bar definitions were specified in the figure legends. Statistical significance was determined using unpaired, two-tailed Student’s t-tests for two groups or one-way analysis of variance (ANOVA) with Dunnett’s multiple comparison test for multiple groups. *P < 0.05, **P < 0.01, ***P < 0.001, ****P < 0.0001 were considered statistically significant. All data analysis was conducted in Prism 8 (GraphPad).

## Supplementary Material

Support file
