# Supplementary material for: LPA1 antagonist-derived LNPs deliver A20 mRNA and promote anti-fibrotic activities
Source: Nano Res. Author manuscript; Available in PMC 2025 Jul 31. (PMC12311867; doi:10.1007/s12274-024-6747-6)
Supplement: Support file [file NIHMS2036499-supplement-Support_file.pdf]

Electronic Supplementary Material

LPA$_1$ antagonist-derived LNPs deliver A20 mRNA and promote anti-fibrotic activities

Jingyue Yan[1,§], Diana D. Kang [1,2,§], Chang Wang [2,§], Xucheng Hou[2], Shi Du[1], Siyu Wang[2], Yonger Xue[1,2], Zhengwei Liu[2], Haoyuan Li[2], Yichen Zhong[2], Binbin Deng[3], David W. McComb[3,4], and Yizhou Dong[1,2] (✉)

[1] *Division of Pharmaceutics & Pharmacology, College of Pharmacy, The Ohio State University, Columbus, Ohio 43210, USA*
[2] *Icahn Genomics Institute, Precision Immunology Institute, Department of Immunology and Immunotherapy, Department of Oncological Sciences, Tisch Cancer Institute, Biomedical Engineering and Imaging Institute, Friedman Brain Institute, Icahn School of Medicine at Mount Sinai, New York, NY 10029, USA*
[3] *Center for Electron Microscopy and Analysis, The Ohio State University, Columbus, Ohio 43212, USA*
[4] *Department of Materials Science and Engineering, The Ohio State University, Columbus, Ohio 43210, USA*
[§] *Jingyue Yan, Diana D. Kang, and Chang Wang contributed equally to this work.*

Supporting information to https://doi.org/10.1007/s12274-024-6747-6

Supplementary methods

Synthesis of LPA$_1$-angonist derived lipids

All LPA$_1$-angonist derived lipids were purified by column chromatography using a CombiFlash Rf system with a RediSep Gold Resolution silica column (Teledyne Isco) with gradient elution. All ^{1}H NMR spectra were run on a Bruker Avance 400 MHz instrument. Mass spectrometric measurements were performed by Acquity SQD UPLC/MS (Waters) or microflex LRF MALDI-TOF mass spectrometer (Bruker) at Icahn School of Medicine at Mount Sinai.

Aldehydes and [1-R] were synthesized according to previously reported procedures[1].

To a solution of LPA$_1$-angonist AM095 or AM966 (0.1 mmol), 3 mL of THF and

Address correspondence to yizhou.dong@mssm.edu

Triphenylphosphine (0.3 mmol), diethyl azodicarboxylate (DEAD, 0.3 mmol) was added dropwise. The solution was stirred for 10 min at 0 °C, [1-R] (0.1mmol) was added, then kept stirred at room temperature overnight. The resulting mixture was diluted with DCM, washed three times with brine (50 mL), and dried over anhydrous Na_2SO_4. After the solvent was removed under reduced pressure, the residue was further purified by Combiflash column chromatography using a silica column (Buchi) with gradient elution from 100% CH_2Cl_2 to 10% CH_2Cl_2/MeOH/NH_4OH (75/25/3, v/v/v) to give compound LPA_1-antagonist derived lipids.

LL1 (30 mg, 29%): ^{1}H NMR (400 MHz, $CDCl_3$) δ 7.76 (m, 2H), 7.69 – 7.58 (m, 3H), 7.54 (m, 2H), 7.49 –7.18 (m, 6H), 6.21 (q, 1H), 4.26 – 4.09 (t, 2H), 3.69 (s, 2H), 2.73 (m, 4H), 2.57 – 2.35 (m, 4H), 2.27 (s, 3H), 1.78-1.33 (m, 63H), 0.87 (t, *J* = 6.7 Hz, 9H). MS for $C_{67}H_{106}N_4O_5$ ($[M+H]^+$) Calculated: 1047.8, Found: 1047.8.

LL2 (25 mg, 22%): ^{1}H NMR (400 MHz, $CDCl_3$) δ 7.75 – 7.55 (m, 7H), 7.49 –7.18 (m, 6H), 6.21 (q, 1H), 4.65 (s, 6H), 4.26 – 4.09 (t, 2H), 3.69 – 3.50 (m, 14H), 2.73 (m, 4H), 2.57 – 2.35 (s, 3H), 2.27 (m, 4H), 1.78-1.33 (m, 72H), 0.88 (t, *J* = 6.7 Hz, 9H). MS for $C_{70}H_{112}N_4O_{11}$ ($[M+H]^+$) Calculated: 1185.8, Found: 1185.8.

LL3 (28 mg, 23%): ^{1}H NMR (400 MHz, $CDCl_3$) δ 7.75 – 7.55 (m, 7H), 7.49 –7.18 (m, 6H), 6.21 (q, 1H), 4.66 (q, *J* = 5.6 Hz, 3H), 4.20 (m, 2H), 3.70 – 3.39 (m, 14H), 2.70 (m, 4H), 2.57 – 2.35 (m, 4H), 2.21 (s, 3H), 1.78-1.33 (m, 70H), 0.87 (t, *J* = 6.7 Hz, 9H). MS for $C_{73}H_{118}N_4O_{11}$ ($[M+H]^+$) Calculated: 1227.9, Found: 1227.9.

LL4 (38 mg, 35%): ^{1}H NMR (400 MHz, $CDCl_3$) δ 7.95 – 7.75 (m, 2H), 7.69 – 7.58 (m, 3H), 7.54 (m, 2H), 7.47 – 7.29 (m, 5H), 6.20 (q, 1H), 4.38 – 4.21 (m, 2H), 3.77 – 3.60 (s, 2H), 2.68 (m, 4H), 2.29 (m, 4H), 2.27 (s, 3H), 1.59-1.03 (m, 69H), 0.89 (t, *J* = 6.7 Hz, 9H). MS for $C_{67}H_{105}ClN_4O_5$ ($[M+H]^+$) Calculated: 1081.8, Found: 1081.8.

LL5 (23 mg, 19%): ^{1}H NMR (400 MHz, $CDCl_3$) δ 7.75 – 7.52 (m, 7H), 7.49 –7.33 (m, 5H), 6.24 (q, 1H), 4.65 (s, 6H), 4.18 (m, 6H), 3.65 – 3.42 (m, 12H), 2.87 – 2.37 (m, 8H), 2.24 (s, 3H), 1.78 – 1.04 (m, 65H), 0.87 (t, *J* = 6.7 Hz, 9H). MS for $C_{70}H_{111}ClN_4O_{11}$($[M+H]^+$) Calculated: 1219.8, Found: 1219.8.

LL6 (27 mg, 22%): ^{1}H NMR (400 MHz, $CDCl_3$) δ 7.75 – 7.52 (m, 7H), 7.49 –7.33 (m, 5H), 6.24 (q, 1H), 4.66 (q, *J* = 5.6 Hz, 3H), 4.21 (m, 6H), 3.70 – 3.42 (m, 14H), 2.80 – 2.35 (m, 8H), 2.21 (s, 3H), 1.78 – 1.04 (m, 68H), 0.88 (t, *J* = 6.7 Hz, 9H). MS for $C_{73}H_{117}ClN_4O_{11}$($[M+H]^+$) Calculated: 1261.8, Found: 1261.8.

Table S1 N/P ratio of LA-LNPs

N/P ratio (mol/mol)

	10:1 weight ratio of LA:mRNA	
	**LA mol. Wt.**	**N/P ratio**
**LA1**	1046.81	6.5
**LA2**	1184.83	5.7
**LA3**	1226.88	5.5
**LA4**	1080.78	6.3
**LA5**	1218.79	5.6
**LA6**	1260.84	5.4

Table S2 Design of orthogonal table L_{16}

Design of Orthogonal Table $L_{16'}$

Exp. No.	Factors (molar ratio)			
	**LA5**	**DOPE**	**Chol**	**PEG**
**LA5-1**	20	20	30	0.5
**LA5-2**	20	30	40	0.75
**LA5-3**	20	40	50	1
**LA5-4**	20	50	60	1.25
**LA5-5**	30	20	40	1
**LA5-6**	30	30	30	1.25
**LA5-7**	30	40	60	0.5
**LA5-8**	30	50	50	0.75
**LA5-9**	40	20	50	1.25
**LA5-10**	40	30	60	1
**LA5-11**	40	40	30	0.75
**LA5-12**	40	50	40	0.5
**LA5-13**	50	20	60	0.75
**LA5-14**	50	30	50	0.5
**LA5-15**	50	40	40	1.25
**LA5-15**	50	50	30	1

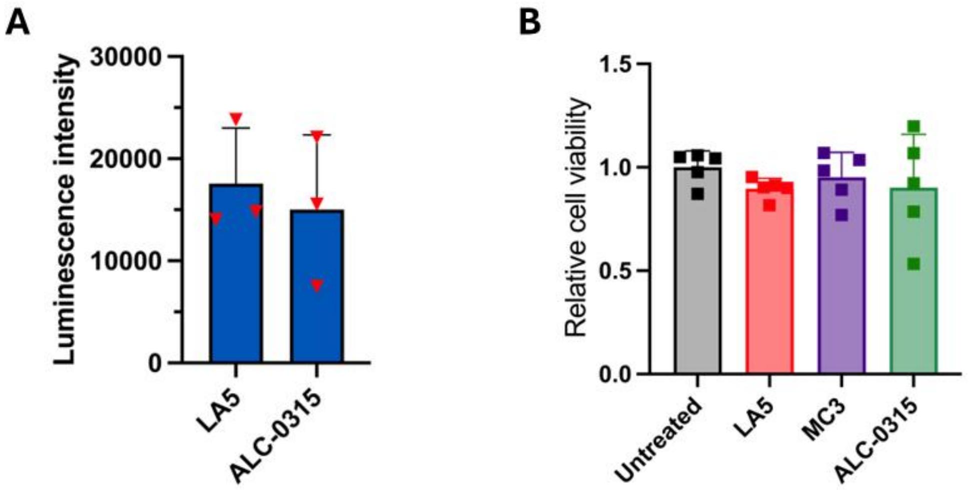

Figure S1 LA5 LNPs characterization. (A) Luminescence intensity in MLg cells after treatment with LA5 or ALC-0315 LNPs with firefly luciferase mRNA. (B) Relative cell viability of isolated primary mouse lung fibroblasts after treatment with LA5, MC3, or ALC-0315 LNPs with firefly luciferase mRNA. Relative cell viability measured with an MTT assay. Data in A and B are presented as the mean ± S.D. (n = 3).

Reference

1. Zhang, Y.; Yan, J.; Hou, X.; Wang, C.; Kang, D. D.; Xue, Y.; Du, S.; Deng, B.; McComb, D. W.; Liu, S.-L.; Zhong, Y.; Dong, Y., STING Agonist-Derived LNP-mRNA Vaccine Enhances Protective Immunity Against SARS-CoV-2. *Nano Letters* **2023,** *23* (7), 2593-2600.
